# Case of Axillary Aneurism, in Which a Ligature Was Applied to the Subclavian Artery, and in Which a Gangrenous Condition, Both of the Upper and Lower Extremities, Supervened

**Published:** 1827-06

**Authors:** 

**Affiliations:** St. George's Hospital.


*Case of Axillary Aneurism, in which a Ligature was applied to the Subclavian Artery, and in which a Gangrenous Condition, both of the Upper and Lower Extremities, supervened.* Treated by Mr. Brodie, at St. George's Hospital.

John Herbert, æt. fifty-six, a countryman, of florid complexion and good constitution; admitted March 4th, 1823. He has had aneurism of the axillary artery on the left side about two months. It occurred spontaneously, and, a fortnight after he had observed a small swelling, the pulsation became perceptible in the tumor. He has had very little pain, and has suffered scarcely any inconvenience from the swelling, which is about the size of a hen's egg, or rather larger, of an oblong shape, in the direction of the artery; the tumor gradually fading off at each end, the surface being smooth and nearly uniform, soft and partly compressible, not painful to the touch; situated just at the edges of the pectoralis and latissimus dorsi, so that the whole of the tumor can be felt.

He was bled, March 5th, to eight ounces, and took some opening medicine.

March 7th.—Soon after twelve the operation was performed. An incision was made along the upper edge of the clavicle, and one small artery was tied. The external jugular vein was tied in two places, and divided between the two ligatures: one of them slipped off afterwards, but this did not cause much bleeding. The subclavian artery was then tied, by means of Weiss's needle, with a ligature of two threads. The operation was performed with great facility, the whole of it being completed in twelve minutes and a half. The pulse at the wrist was immediately stopped; the power of motion was not at all impaired; and he complained of no numbness, though a sensation was felt to extend down the arm when the nerves were touched in the operation. After the effects of exposure had gone off, the warmth of the arm was natural, and appeared to be exactly equal to that of the other: it was, however, wrapped in flannel.

Vespere.—The tumor diminished to half its former size, and apparently harder. Complains of pain in the bowels.

℞. Pulv. Ant. gr. iij.; Exr. Papav. alb. gr. vj. M. h. s. s.

March 8th.—Arm warm; no pain in the shoulder.

Haust. Sennæ bis.—℞. Haust. Salin. ℥jss.; Liq. Ant. Tart. m. xij. M. sextis horis.

Vespere.—Tongue rather furred, and dry; skin heated, and not moist; pulse ninety-four. He has suffered considerably from pain in the bowels, which have not been open since the 6th.

Enema commune statim.

The glyster was repeated at eleven P.M.; after which he had one motion, and was much relieved.

9th, (*second day.*)—Has not had a very quiet night, on account of the irritability of the bowels. Tongue thickly coated with brown fur, with white edges; breath fetid; head heavy and dull; no pain in the arm. Pulse 104, full, and rather hard. There has been a good deal of oozing from the wound.

Repetatur Enema, nine A.M.

Vespere.—Much relieved by three motions. He has been in a profuse perspiration during the afternoon. Pulse ninety-eight, soft.

At half-past twelve at night, he was suddenly seized with acute burning pain, extending down the whole of the arm to the palm of the hand, lasting three or four minutes, and followed by a rather severe rigor of twenty minutes' duration. He took twenty drops of laudanum. After the rigor he felt hot and dry before the profuse perspiration returned.

10th, (*third day.*)—He passed a pretty comfortable night after the perspiration came on. Tongue foul. Pulse eighty-four, soft, and regular. The wound was dressed with dry lint: it was sloughy, the sloughs being of a dark colour, and affecting part of the skin as well as the cellular membrane; with a slight inflammatory blush surrounding the wound. Still, however, he has had scarcely any pain, and the affected limb has been warm the whole time.

Haust. Sennæ mane.

11th, (*fourth day.*)—Has had a pretty good night, without any more shivering. Bowels still uncomfortable; and he complains of his medicine. Omitt^r Haust. Pulse ninety-two, full, but not very hard. Nine A.M. V.S. ad ℥vj. Blood highly buffed, but not at all cupped. The wound was dressed with T. Benz. C. though sloughs did not appear to be extending, nor was the shoulder painful.

Soon after the dressing, another rigor came on, (at half-past two P.M.) with slight pain and numbness of the arm. It lasted about a quarter of an hour, but scarcely checked the copious perspiration in which he has lain since the afternoon of the 9th.

Six P.M.—The pulse having risen in force, though not in frequency, eight ounces of blood were taken, (ordered when the rigor came on.) This blood also was much buffed, but not cupped. Bowels open twice. His right elbow has become much swollen, tender and painful, apparently from rheumatic inflammation, to which he is subject.

12th, (*fifth day.*)—Has had a very restless and unquiet night, not relieved by two doses of laudanum, of forty drops each. He had a third shivering fit at three o'clock; has been rather delirious in the night, and has tried to rise from bed, moving his arm considerably. A fatal change has taken place in his countenance, which is sunk and anxious. Pulse 120, small, and irritable; perspiration continues; arm warm as usual; tongue not so brown, but still loaded and clammy. Thirst increased.

Nine A.M.—℞. Pulv. Ant. gr. iv.; Opii gr. ij. M. statim.

One P.M.—He has slept a good deal, and is much under the influence of the opium. Is now sensible, but his thoughts wander when he is left to himself. Wound not more sloughy, but of a darker colour, and the sloughs do not seem likely to separate.

Linseed poultice applied.—℞. Calomel gr. ij.; Opii gr. j. statim, et h. s. repet.—℞. Mist. Camph. ℥ jss.; Træ. Digitalis m. x.; Træ. Opii gtt. v. M. quartis horis.

Nine P.M.—He has sunk considerably, and the countenance is still more alarming. Pulse 140 or 150, weak, and unsteady.

℞. Ammon. Carbon. gr. xx.; Aquæ ℥ j. M. secundis horis, cum Suc. Limonis recentis ℥ ss.—Omit. Mist. c. Digital.

He continued to sink gradually till two A.M. March 13th, when he died, five days and a half after the operation.

The body after death had the appearance of gangrene, the whole of the right arm, part of the left, and part of both legs, becoming quite livid and soft; and, on cutting into them, the muscles and other textures were found to be soft and putrid. The inner surface of the trachea also had a livid colour.

The subclavian artery was found to be plugged up for about half an inch on each side of the ligature, which had begun to ulcerate through the vessel. The inner surface of the aorta was rather more red than natural, and the subclavian vein was of a dark colour. No disease was found in the heart, nor in any of the large arteries; nor was any other appearance detected to which the death could be attributed.

It is remarkable that the gangrenous appearance was less distinct in the arm of which the artery had been tied, than in the other limbs.

